# The Silent Burden: Understanding Alexithymia and Its Correlation With University Student Depression, Anxiety, and Stress in a Cross‐Sectional Study

**DOI:** 10.1002/hsr2.70677

**Published:** 2025-08-27

**Authors:** Asma Darvishi, Nikoo Almani, Fatemeh Zarimeidani, Rahem Rahmati, Hadi Raeisi Shahraki

**Affiliations:** ^1^ Students Research Committee Shahrekord University of Medical Sciences Shahrekord Iran; ^2^ Student Research Committee Shahid Sadoughi University of Medical Sciences Yazd Iran; ^3^ Department of Epidemiology and Biostatistics, Faculty of Health Shahrekord University of Medical Sciences Shahrekord Iran

**Keywords:** medical students, mental disorder, mood disorder, psychiatric, psychology

## Abstract

**Background and Aims:**

In a time of increasing mental health difficulties among college students globally, where 20%–45% experience disorders annually, mood disorders emerge as a prevalent concern. These disorders mainly impact individuals aged 18 to 30 and significantly affect academic performance and long‐term well‐being. This study aimed to assess the psychological well‐being of university students, focusing on alexithymia, depression, anxiety, stress, and their complex relationships.

**Methods:**

Using a multi‐stage sampling approach, the study was conducted in 2019 with 260 undergraduate students at Shahrekord University of Medical Sciences, Shahrekord, Iran. The Depression, Anxiety, and Stress Scale (DASS) questionnaire measured depression, anxiety, and stress, while the Toronto Alexithymia Scale‐20 (TAS‐20) assessed alexithymia. The data were analyzed using SPSS v.23.0.

**Results:**

The participants had a mean age of 20.7 ± 3.2 years, were mostly female (75.7%), single (90.7%), and of Fars ethnicity (66.1%). The majority lived in dormitories (70.3%). Alexithymia was present in 30.8% of the population, with males scoring higher than females (*p* = 0.04). Also, students aged 18–19 had lower depression (*p* = 0.04) and anxiety (*p* = 0.04) scores. We found significant positive correlations between alexithymia and stress, depression, and anxiety (*p* < 0.001). Moreover, a strong positive correlation was observed between depression and both anxiety (*p* < 0.001) and stress (*p* < 0.001). Additionally, anxiety demonstrated a notable correlation with stress (*p* < 0.001), underscoring the intricate interplay among these psychological factors.

**Conclusion:**

Identifying alexithymia in medical settings is essential, as it can affect patient–provider communication and care. Students with alexithymic traits may benefit from interventions targeting both alexithymia and co‐occurring mental disorders like depression and anxiety. Future research should focus on developing tailored treatments and early screening to improve emotional regulation and mental health outcomes, particularly in high‐stress academic environments.

## Summary

- Approximately one in three university students showed alexithymia traits.
- Alexithymia, depression, anxiety, and stress were significantly associated with each other in university students.
- Between university students, sex was significantly associated with alexithymia, and age with depression and anxiety.

## 1 Introduction

According to several studies, 20%–45% of college students encounter at least one mental health condition in any 1 year, and mental diseases are becoming more prevalent in colleges worldwide [1, 2]. Most commonly, the development and significant incidence of common mental health conditions, such as mood disorders, happen in people between 18 and 30, particularly among students [1, 3].

Depressive disorders are the most common global cause of disability [3]. University students with noticeable workloads are more likely to experience depression [4]. According to meta‐analysis, students are even more likely than others to face mild to extremely severe depressive symptoms, with one in three experiencing them [5, 6]. Regarding that, Asian and younger students were more depressed [6]. Regrettably, a meta‐analysis of Iranian university students revealed that nearly half of them experience depression—a notably escalating trend [7].

Among the most prevalent mental illnesses, anxiety disorders significantly negatively influence people's quality of life and have a high social and financial burden [8]. Anxiety affects around one in three students worldwide, significantly more than the overall population [9]. Besides, high stress among college and recently graduated students is an alarming public health issue. In this regard, university students report more chronic stress than the general population, negatively affecting their emotional, academic, and physical well‐being [8, 10].

There is evidence that people with mental and psychosomatic diseases have a high prevalence of alexithymia [11]. Alexithymia is a complex personality trait defined by challenges in recognizing and articulating emotions and a tendency toward externally focused thinking. It is characterized by difficulty discriminating between bodily sensations and feelings, struggles expressing emotions to others, and a cognitive approach with attention on the outside [12, 13].

While the general population's prevalence of alexithymia is between 15% and 20%, it is more significant in clinical populations [14]. However, among university students in Iran, the prevalence of alexithymia stands at 21.8%–43.8% in different studies [15, 16], surpassing that of the general population samples. Shahrekord, Iran, presents a particularly relevant but under‐researched setting for studying alexithymia and mental health among university students. Its high‐altitude climate, seasonal extremes, and conservative cultural attitudes toward mental health may influence students' psychological well‐being. Since most research focuses on metropolitan areas, Shahrekord remains largely overlooked, highlighting the need to study alexithymia in this region to better understand local mental health challenges and create effective interventions.

In this light, alexithymic persons, particularly students, tend to engage in disruptive behaviors like suicidal thoughts and actions, drug misuse, disappointing academic performance, and poor self‐care, and feel more isolated and misaligned with campus life [13, 17, 18, 19]. Research also found that even after controlling for confounding factors, persons with anxiety or depressive disorders are still likely to have greater alexithymia levels than normal controls [20]. Nevertheless, there is an ongoing discussion on whether alexithymia is a risk factor, a side effect, or a co‐occurring condition with depression or anxiety [13, 20].

Exploring alexithymia and its connections among university students could prove advantageous in designing effective intervention programs for the early identification and treatment of alexithymia and related disorders.

## 2 Methods

### 2.1 Study Design

This cross‐sectional study was conducted among 260 Shahrekord University of Medical Sciences (SKUMS) undergraduate students in 2019 in Shahrekord, Iran. This study project was approved by the Ethics Committee of SKUMS (Ethics code: IR.SKUMS.REC.1398.108). Multi‐stage cluster sampling was used to select our sample from various faculties. Written informed consent was obtained from the participants. The study included undergraduate students aged 18–30 who provided self‐consent, had no history of psychological or medical history, and had not experienced any stressful events like the death of a close family member, divorce, a major health concern, illness, or injury in the past month. Students who did not fully complete the questionnaires were excluded from the study. The objective of this study was to determine the average scores of alexithymia, depression, anxiety, and stress among university students, and investigate any potential associations between these four conditions. The sample size calculation was performed based on a previous study considering the correlation coefficient of 0.34 between depression and alexithymia, the type one error of 0.001, and the type two error of 0.01 [21].

### 2.2 Data Collection

#### 2.2.1 Depression, Anxiety, and Stress

The Depression, Anxiety, and Stress Scale (DASS) is a self‐administered tool that is capable of distinguishing between symptoms of depression, anxiety, and stress in either clinical or nonclinical samples. It is a standardized instrument that is commonly used in a variety of circumstances [22]. It has been verified that the questionnaire has appropriate psychometric qualities and is equivalent to other reliable measures [23].

Respondents are tasked with answering 21 items on the scale that center on whether they have experienced depression, anxiety, and stress. The depression section assesses a loss of drive and self‐worth. The anxiety subscale mainly assesses signs of ongoing worry and anxiety. The stress subscale addresses persistent arousal and irritation symptoms. Questions 1 through 7 assess depressive symptoms, questions 8 through 14 assess anxiety symptoms, and questions 15 through 21 assess stress symptoms. Using a Likert scale with a maximum of four points, 0 indicates “did not pertain to me on any level,” 1 indicates “applied to me to some extent, “2” indicates that “applied to me in a significant way,” and 3 means “ applied to me frequently, if not always.” Scores below 9, 7, and 14 are regarded as “normal” for depression, anxiety, and stress, respectively.

The questionnaire's general Cronbach's alpha reliability score was 0.94, while the values for the depression and anxiety components were 0.87 and 0.89, respectively. However, it was observed to be somewhat lower for the stress dimension [24].

#### 2.2.2 Alexithymia

Toronto Alexithymia Scale, created by Taylor and Bagby [25], is the most popular self‐assessment indicator for alexithymia. This scale assesses five dimensions, (I) incapacity to distinguish between physical signs of emotional arousal and sentiments and to identify them; (II) problems sharing sentiments with others; (III) lack of introspection or externally focused thinking; (IV) social conformism; and (V) absence of daydreaming or other forms of imaginative activity.

Nearly concurrently, the authors offered the TAS‐20, a somewhat condensed version with more potent psychometric qualities than the preceding TAS series iterations. Twenty‐five years since its introduction, the TAS‐20 is still the common determiner. Only the first three traits were retained as criteria on this scale. The final two traits continue in components 2 and 3 as a more extensive operatory thinking component concentrated on the choice of thought material acquired from everyday life's external features over those gained from interior experience [26].

The TAS‐20's internal consistency reliability has been reported as Cronbach = 0.79 in a standard sample and test–retest = 0.77 in a clinical sample [27]. Ample levels of convergent and concurrent validity have also been shown in research using the TAS‐20 [28].

A 5‐point Likert scale is used to ask the respondents their opinions, expressing their level of consensus or disagreement with each claim. Overall ratings are between 20 and 100. Scores of 61 or above indicate Alexithymia, and scores between 52 and 60 points to the possible problem [29].

### 2.3 Statistical Analysis

The statistical analysis was performed with SPSS 23.0 software, using the Pearson correlation (to assess the correlation between two quantitative variables), independent *t*‐test (to compare means between two groups), and ANOVA test (to compare means among more than two groups). *p* < 0.05 was considered as statistically significant. All of the tests were two‐sided.

## 3 Results

In this study, 260 students met the inclusion criteria. The mean ± SD of participants' age was 20.7 ± 3.2 years. The majority of students were female (75.7%) and single (90.7%), had Fars ethnicity (66.1%), and were in dormitory (70.3%). According to the TAS‐20, 80 out of 260 students (30.8%) displayed signs of alexithymia.

The alexithymia score was significantly higher among male students (55.4 ± 12.6 vs. 51.6 ± 12.5, *p* = 0.04). Also, the score of depression was lower in younger students aged between 18 and 19 (4.3 ± 3.2 vs*.* 5.3 ± 3.8 (age ≥ 20), *p* = 0.04). Similarly, the anxiety score was lower in younger students aged between 18 and 19 (5.6 ± 4.5 vs. 6.9 ± 5.1, *p* = 0.04). Other demographic characteristics, including marital status, ethnicity, and residence in the dormitory, had no significant association with alexithymia, depression, anxiety, and stress (Table 1).

**Table 1:** Association of demographic characteristics with alexithymia, depression, anxiety, and stress.

Variable	Alexithymia	Depression	Anxiety	Stress
Sex	*p* = 0.04 D = 0.30	*p* = 0.31 D = 0.16	*p* = 0.66 D = 0.08	*p* = 0.25 D = 0.16
Female (*n* = 197)	51.6 ± 12.5	4.8 ± 3.7	6.4 ± 5.0	5.4 ± 4.4
Male (*n* = 63)	55.4 ± 12.6	5.4 ± 3.4	6.8 ± 4.7	6.1 ± 4.1
Age	*p* = 0.31 D = 0.13	*p* = 0.04 D = 0.28	*p* = 0.04 D = 0.29	*p* = 0.07 D = 0.28
18–19 (*n* = 83)	53.7 ± 13.3	4.3 ± 3.2	5.6 ± 4.5	4.8 ± 3.9
20 ≤ (*n* = 177)	52.0 ± 12.3	5.3 ± 3.8	6.9 ± 5.1	5.9 ± 4.5
Marital status	*p* = 0.82 D = 0.0.05	*p* = 0.77 D = 0.08	*p* = 0.11 D = 0.37	*p* = 0.23 D = 0.27
Single (*n* = 236)	52.5 ± 12.5	4.9 ± 3.6	6.7 ± 5.0	5.7 ± 4.3
Married (*n* = 24)	53.1 ± 13.9	5.2 ± 4.0	5.0 ± 4.1	4.5 ± 4.7
Ethnicity	*p* = 0.38 D = 0.31	*p* = 0.67 D = 0.11	*p* = 0.73 D = 0.11	*p* = 0.91 D = 0.06
Fars (*n* = 172)	51.9 ± 12.5	5.1 ± 3.7	6.7 ± 4.9	5.6 ± 4.3
Lor (*n* = 58)	52.9 ± 12.7	4.6 ± 3.6	6.2 ± 4.9	5.6 ± 4.7
Other (*n* = 30)	55.3 ± 12.8	4.9 ± 3.3	6.1 ± 5.1	5.2 ± 4.9
Residence in dormitory	*p* = 0.50 D = 0.10	*p* = 0.77 D = 0.05	*p* = 0.66 D = 0.06	*p* = 0.30 D = 0.14
Yes (*n* = 183)	52.9 ± 12.7	4.9 ± 3.5	6.6 ± 5.0	5.7 ± 4.5
No (*n* = 77)	51.7 ± 12.4	5.1 ± 4.0	6.3 ± 4.7	5.1 ± 4.0

**Figure 1:**
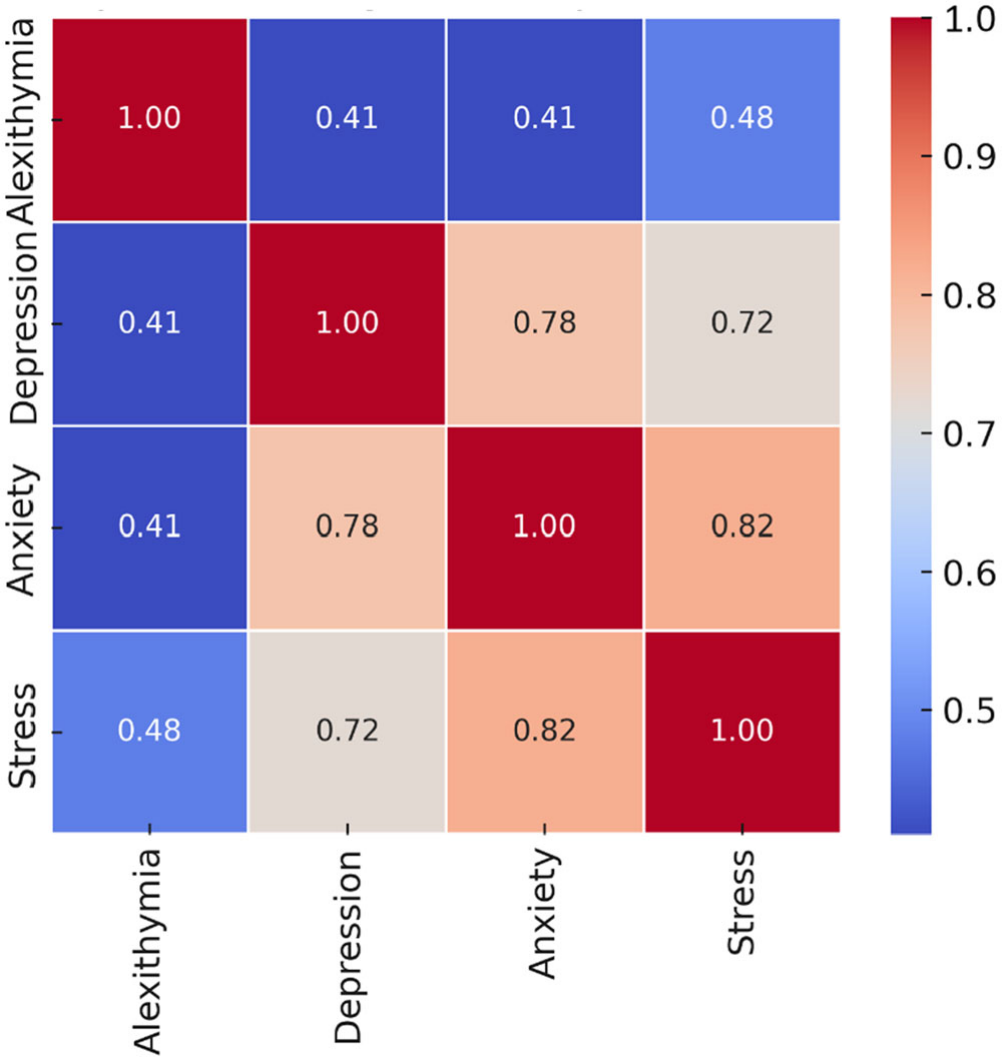
The Pearson correlations between alexithymia, depression, anxiety, and stress.

The results obtained from Pearson correlation showed positive correlations between alexithymia and stress, depression, and anxiety (*p* < 0.001). Depression was positively correlated with both anxiety (*p* < 0.001) and stress (*p* < 0.001). Also, anxiety was correlated with stress (*p* < 0.001) (Figure 1).

## 4 Discussion

Alexithymia is one of the issues that students encounter, and they are not well informed about it. Our research intended to determine how alexithymia, stress, and anxiety are related while also considering variables such as age, sex, and dorm living. According to the findings of the correlational study, stress, depression, and anxiety are all positively correlated with alexithymia. Additionally, we found a strong correlation between sex and alexithymia. However, no correlation was found between alexithymia and other features, including age, marital status, ethnicity, or living in the dormitory.

Clinically, it is evident that there is still much to learn about alexithymia and its association with various related conditions. Many sociodemographic characteristics, such as male sex, have been linked to alexithymia in earlier research concentrating on general populations [30, 31]. In highly masculine cultures, men exhibit greater alexithymia than women. Since societal expectations frequently encourage emotional repression in males, which may make it harder for them to detect and express their feelings, gender socialization and cultural standards may contribute to the development of alexithymia in men [30]. Comparative studies in Western societies, where emotional expression is less stigmatized, indicate lower alexithymia prevalence among men. This suggests that cultural attitudes towards emotional expression significantly shape alexithymia levels across different regions.

While there was no correlation between the prevalence of alexithymia and age in our study as others [32, 33], TAS‐20 scores and age have been found to correlate positively in an earlier study [31]. In another study, alexithymia had also been associated with aging, including the overall population in the 30–97 age spectrum [34]. Respondents in our research were split into two age groups: those 18‐ to 19‐years‐old and individuals at least 20 years old. All questionnaire respondents were under the age of 30. One explanation for this discrepancy could be the age group of the two investigations. It is of note that alexithymia can happen at any age. Depending on other elements like personality traits and life experiences, there may be a relationship between age and alexithymia.

Similar to the findings of other studies [14, 17, 35], we found a strong correlation between depression and alexithymia. Until now, numerous theories have been put forth about the connection between depression and alexithymia. Most research also favors the vulnerability theory that alexithymia makes people prone to depression [17]. A prospective study revealed that a high baseline alexithymia score greatly anticipated depression at follow‐up [36]. Due to the high correlation between alexithymia and depression, depression must be included as a confounding factor when investigating alexithymia in general populations [37].

The results of this study corroborate prior studies showing a connection between alexithymia and anxiety [14, 35]. This may be because alexithymic individuals have difficulty recognizing their emotional state, leading to emotional confusion and dysregulation, which can contribute to anxiety. Several studies have also shown that anxiety seems to have a direct impact on alexithymia. However, depression only seems to have an indirect impact through anxiety [38, 39].

Consistent with other studies, our findings demonstrated a connection between stress and alexithymia and the insufficiency of coping mechanisms with stress [40, 41]. The fact that people with alexithymia have inadequate emotional intelligence lends credence to this notion, which, combined with difficulty understanding and explaining feelings, decreases their capacity to deal with stressful situations. A positive correlation exists between alexithymia and stress since an individual's incapacity to handle challenging circumstances puts them under a lot of stress. Additionally, stress can exacerbate alexithymia and various other symptoms of illnesses [6]. Anxiety and other unfavorable feelings may be brought on by stressful circumstances, which can make it even more challenging to recognize and express destructive emotions.

The findings of this study suggest that universities should implement mental health interventions for students with alexithymic traits, focusing on emotional literacy, stress management, and cognitive‐behavioral strategies. Counseling services should also recognize these traits and offer targeted support. Integrating emotional intelligence training into the curriculum could improve self‐awareness and emotional regulation. By gaining a deep insight into students' needs and preferences, healthcare providers can tailor educational programs to address those needs effectively, leading to improved overall results [42].

Due to the paucity of research on alexithymia in Iranian communities, particularly among university students who experience high academic and social pressures, further investigation is necessary. Future research can help determine the prevalence of alexithymia in different segments of Iranian society and identify populations at higher risk. Cross‐cultural comparisons may offer valuable insights into the extent to which cultural norms influence alexithymia and its related conditions. Given the impact of alexithymia on mental health, educational institutions and policymakers should take proactive steps to address emotional awareness deficits and promote mental well‐being among students.

### 4.1 Limitations

This study has a number of limitations. The research sample was made up of students chosen from a city in Iran; greater variation in the sample's age distribution and other cultural and socioeconomic variables would have increased the validity of the findings. It also restricts the generalizability of the findings to other regions or populations. Moreover, using cross‐sectional data to build the network prevents us from identifying causal links. The study relies on self‐reported data, which can introduce biases, especially when assessing alexithymia. Given that alexithymia involves difficulty in identifying and expressing emotions, individuals may struggle to accurately report their emotional experiences, leading to potential underreporting or misreporting. Future research should incorporate objective methods, such as clinician evaluations or observer reports, to reduce bias.

## 5 Conclusion

The study highlighted a substantial correlation between alexithymia, depression, anxiety, and stress among university students. The findings revealed a significant correlation between alexithymia and higher levels of depression, anxiety, and stress. Males showed higher levels of alexithymia, and younger students (aged 18–19) exhibited lower depression and anxiety scores. These results highlight the need for interventions focused on improving emotional regulation, particularly for students with alexithymic traits who may also experience co‐occurring mental disorders. Therefore, it becomes even more imperative to contemplate the implementation of regular screenings for these psychological issues among university students and intervene promptly to mitigate potential complications. Further research should focus on developing early screening tools for alexithymia in high‐stress academic environments. Also, extensive cohort studies are essential to ascertain the presence of causal relationships among these conditions or their mutual exacerbation.

## Author Contributions

**Asma Darvishi:** investigation and resources. **Nikoo Almani:** writing – original draft, writing – review and editing. **Fatemeh Zarimeidani:** writing – original draft, writing – review and editing. **Rahem Rahmati:** project administration, writing – review and editing and methodology. **Hadi Raeisi Shahraki:** conceptualization, supervision, methodology, data curation, formal analysis, and validation.

## Ethics Statement

The Ethics Committee of Shahrekord University of Medical Sciences confirmed the protocol of this study (IR.SKUMS.REC.1398.108). Written informed consent was obtained from the participants. The study was conducted in accordance with Helsinki's declaration.

## Conflicts of Interest

The authors declare no conflicts of interest.

## Transparency Statement

The lead author Hadi Raeisi Shahraki affirms that this manuscript is an honest, accurate, and transparent account of the study being reported; that no important aspects of the study have been omitted; and that any discrepancies from the study as planned (and, if relevant, registered) have been explained.

## Data Availability

All authors have read and approved the final version of the manuscript. Hadi Raeisi Shahraki had full access to all of the data in this study and took complete responsibility for the integrity of the data and the accuracy of the data analysis. All relevant data were reported in the manuscript. For further information, the corresponding author can be contacted.

## References

[1] Y. Amanvermez , M. Rahmadiana , E. Karyotaki , et al., “Stress Management Interventions for College Students: A Systematic Review and Meta‐Analysis,” Clinical Psychology: Science and Practice 223 (2020): e12342.

[2] R. P. Auerbach , P. Mortier , R. Bruffaerts , et al., “WHO World Mental Health Surveys International College Student Project: Prevalence and Distribution of Mental Disorders,” Journal of Abnormal Psychology 127 (2018): 623–638. 30211576 10.1037/abn0000362 PMC6193834

[3] M. N. Khan , P. Akhtar , S. Ijaz , and A. Waqas , “Prevalence of Depressive Symptoms Among University Students in Pakistan: A Systematic Review and Meta‐Analysis,” Frontiers in Public Health 8 (2021): 603357. 33490022 10.3389/fpubh.2020.603357 PMC7820542

[4] X.‐Y. Lei , L.‐M. Xiao , Y.‐N. Liu , and Y.‐M. Li , “Prevalence of Depression Among Chinese University Students: A Meta‐Analysis,” PLoS One 11 (2016): e0153454. 27070790 10.1371/journal.pone.0153454 PMC4829172

[5] A. A. Mirza , M. Baig , G. M. Beyari , M. A. Halawani , and A. A. Mirza , “Depression and Anxiety Among Medical Students: A Brief Overview,” Advances in Medical Education and Practice 12 (2021): 393–398. 33911913 10.2147/AMEP.S302897 PMC8071692

[6] Y.‐J. Tung , K. K. H. Lo , R. C. M. Ho , and W. S. W. Tam , “Prevalence of Depression Among Nursing Students: A Systematic Review and Meta‐Analysis,” Nurse Education Today 63 (2018): 119–129. 29432998 10.1016/j.nedt.2018.01.009

[7] Z. Jaafari , A. Farhadi , F. Amin Lari , et al., “Prevalence of Depression in Iranian College Students: A Systematic Review and Meta‐Analysis,” Iranian Journal of Psychiatry and Behavioral Sciences 15 (2021): e101524.

[8] M. Çalık , “Determining The Anxiety and Anxiety Levels Of University Students in the COVID 19 Outbreak,” International Journal of Medical Science and Clinical Invention 7 (2020): 4887–4894.

[9] T. Tian‐Ci Quek , W. Wai‐San Tam , B. X. Tran , et al., “The Global Prevalence of Anxiety Among Medical Students: A Meta‐Analysis,” International Journal of Environmental Research and Public Health 16 (2019): 2735. 31370266 10.3390/ijerph16152735 PMC6696211

[10] M. Yusufov , J. Nicoloro‐Santabarbara , N. E. Grey , A. Moyer , and M. Lobel , “Meta‐Analytic Evaluation of Stress Reduction Interventions for Undergraduate and Graduate Students,” International Journal of Stress Management 26 (2019): 132–145.

[11] L. Ricciardi , B. Demartini , A. Fotopoulou , and M. J. Edwards , “Alexithymia in Neurological Disease: A Review,” Journal of Neuropsychiatry and Clinical Neurosciences 27 (2015): 179–187. 25658681 10.1176/appi.neuropsych.14070169

[12] O. Luminet , K. A. Nielson , and N. Ridout , “Cognitive‐Emotional Processing in Alexithymia: An Integrative Review,” Cognition & Emotion 35 (2021): 449–487. 33787442 10.1080/02699931.2021.1908231

[13] S. Guidotti , A. Fiduccia , and C. Pruneti , “Introversion, Alexithymia, and Hostility: A Path Analysis From Personality to Suicidal Ideation Among University Students,” Psychological Reports, ahead of print, April 16, 2024: 00332941241247526. 10.1177/00332941241247526 38623941

[14] S. H. Hamaideh , “Alexithymia Among Jordanian University Students: Its Prevalence and Correlates With Depression, Anxiety, Stress, and Demographics,” Perspectives in Psychiatric Care 54 (2018): 274–280. 28726284 10.1111/ppc.12234

[15] M. Faramarzi and S. Khafri , “Role of Alexithymia, Anxiety, and Depression in Predicting Self‐Efficacy in Academic Students,” Scientific World Journal 2017 (2017): 1–7. 10.1155/2017/5798372 PMC5244027 28154839

[16] M. Rahimi , F. Zarimeidani , H. S. Bahreini , A. Kadkhodaei , R. Rahmati , and H. Raeisi Shahraki , “Can Personality Traits be Linked to the Development of Alexithymia? Findings From a Cross‐Sectional Study,” Iranian Biomedical Journal 28 (2024): 10.

[17] L. Hemming , G. Haddock , J. Shaw , and D. Pratt , “Alexithymia and Its Associations With Depression, Suicidality, and Aggression: An Overview of the Literature,” Frontiers in Psychiatry 10 (2019): 203. 31031655 10.3389/fpsyt.2019.00203 PMC6470633

[18] A. Sarman and S. Tuncay , “The Associations of Parental Attitudes and Peer Bullying With Alexithymia in Adolescents: A Structural Equality Model,” Journal of Pediatric Nursing 73 (2023): e372–e380. 37806855 10.1016/j.pedn.2023.10.003

[19] S. H. Alzahrani , S. Coumaravelou , I. Mahmoud , J. Beshawri , and M. Algethami , “Prevalence of Alexithymia and Associated Factors Among Medical Students at King Abdulaziz University: A Cross‐Sectional Study,” Annals of Saudi Medicine 40 (2020): 55–62. 32026718 10.5144/0256-4947.2020.55 PMC7012024

[20] W. Tang , T. Hu , L. Yang , and J. Xu , “The Role of Alexithymia in the Mental Health Problems of Home‐Quarantined University Students During the COVID‐19 Pandemic in China,” Personality and Individual Differences 165 (2020): 110131. 32518435 10.1016/j.paid.2020.110131 PMC7273169

[21] Y. Gan , F. Tian , X. Fan , et al., “A Study of the Relationship Between Social Support, Depression, Alexithymia and Glycemic Control in Patients With Type 2 Diabetes Mellitus: A Structural Equation Modeling Approach,” Frontiers in Endocrinology 15 (2024): 1390564. 39229377 10.3389/fendo.2024.1390564 PMC11368761

[22] O. Ahmed , R. A. Faisal , S. M. A. H. M. Alim , T. Sharker , and F. A. Hiramoni , “The Psychometric Properties of the Depression Anxiety Stress Scale‐21 (DASS‐21) Bangla Version,” Acta Psychologica 223 (2022): 103509. 35065529 10.1016/j.actpsy.2022.103509

[23] I. Marijanović , M. Kraljević , T. Buhovac , et al., “Use of the Depression, Anxiety and Stress Scale (DASS‐21) Questionnaire to Assess Levels of Depression, Anxiety, and Stress in Healthcare and Administrative Staff in 5 Oncology Institutions in Bosnia and Herzegovina During the 2020 COVID‐19 Pandemic,” Medical Science Monitor 27 (2021): e930812‐1. 33867520 10.12659/MSM.930812 PMC8063632

[24] A. Thiyagarajan , T. G. James , and R. R. Marzo , “Psychometric Properties of the 21‐Item Depression, Anxiety, and Stress Scale (DASS‐21) Among Malaysians During COVID‐19: A Methodological Study,” Humanities and Social Sciences Communications 9 (2022): 220. 35789924 10.1057/s41599-022-01229-x PMC9244484

[25] G. J. Taylor , D. Ryan , and M. Bagby . “Toward the Development of a New Self‐Report Alexithymia Scale,” Psychotherapy and Psychosomatics 44, no. 4 (1985): 191–199. 3837277 10.1159/000287912

[26] M. González‐Arias , A. Martínez‐Molina , S. Galdames , and A. Urzúa , “Psychometric Properties of the 20‐Item Toronto Alexithymia Scale in the Chilean Population,” Frontiers in Psychology 9 (2018): 963. 29946289 10.3389/fpsyg.2018.00963 PMC6005868

[27] M. Mazaheri , “Psychometric Properties of the Persian Version of the Difficulties in Emotion Regulation Scale DERS‐6 & DERS‐5‐ Revised in an Iranian Clinical Sample,” Iranian Journal of Psychiatry 10 (2015): 115–122. 26884788 PMC4752524

[28] R. M. Bagby , J. D. A. Parker , and G. J. Taylor , “The Twenty‐Item Toronto Alexithymia Scale—I. Item Selection and Cross‐Validation of the Factor Structure,” Journal of Psychosomatic Research 38 (1994): 23–32. 8126686 10.1016/0022-3999(94)90005-1

[29] A. N. Da Silva , A. B. Vasco , and J. C. Watson , “Alexithymia and Emotional Processing: A Longitudinal Mixed Methods Research,” Research in Psychotherapy: Psychopathology, Process, and Outcome 21 (2018): 292. 32913756 10.4081/ripppo.2018.292 PMC7451369

[30] J. Mendia , L. N. Zumeta , O. Cusi , et al., “Gender Differences in Alexithymia: Insights From an Updated Meta‐Analysis,” Personality and Individual Differences 227 (2024): 112710.

[31] S. Obeid , M. Akel , C. Haddad , et al., “Factors Associated With Alexithymia Among the Lebanese Population: Results of a Cross‐Sectional Study,” BMC Psychology 7 (2019): 80. 31829280 10.1186/s40359-019-0353-5 PMC6907355

[32] J. Barth , L. Bermetz , E. Heim , S. Trelle , and T. Tonia , “The Current Prevalence of Child Sexual Abuse Worldwide: A Systematic Review and Meta‐Analysis,” International Journal of Public Health 58 (2013): 469–483. 23178922 10.1007/s00038-012-0426-1

[33] C. Bressi , G. Taylor , J. Parker , et al., “Cross Validation of the Factor Structure of the 20‐Item Toronto Alexithymia Scale: An Italian Multicenter Study,” Journal of Psychosomatic Research 41 (1996): 551–559. 9032718 10.1016/s0022-3999(96)00228-0

[34] A. K. Mattila , J. K. Salminen , T. Nummi , and M. Joukamaa , “Age Is Strongly Associated With Alexithymia in the General Population,” Journal of Psychosomatic Research 61 (2006): 629–635. 17084140 10.1016/j.jpsychores.2006.04.013

[35] D. A. Preece , A. Mehta , K. Petrova , P. Sikka , E. Pemberton , and J. J. Gross , “Alexithymia Profiles and Depression, Anxiety, and Stress,” Journal of Affective Disorders 357 (2024): 116–125. 38387670 10.1016/j.jad.2024.02.071

[36] V. Günther , M. Rufer , A. Kersting , and T. Suslow , “Predicting Symptoms in Major Depression After Inpatient Treatment: The Role of Alexithymia,” Nordic Journal of Psychiatry 70 (2016): 392–398. 26935972 10.3109/08039488.2016.1146796

[37] K. Honkalampi , J. Hintikka , A. Tanskanen , J. Lehtonen , and H. Viinamäki , “Depression Is Strongly Associated With Alexithymia in the General Population,” Journal of Psychosomatic Research 48 (2000): 99–104. 10750635 10.1016/s0022-3999(99)00083-5

[38] S. Berthoz , S. Consoli , F. Perez‐Diaz , and R. Jouvent , “Alexithymia and Anxiety: Compounded Relationships? A Psychometric Study,” European Psychiatry 14 (1999): 372–378. 10683621 10.1016/s0924-9338(99)00233-3

[39] M. Karukivi , L. Hautala , O. Kaleva , et al., “Alexithymia Is Associated With Anxiety Among Adolescents,” Journal of Affective Disorders 125 (2010): 383–387. 20303180 10.1016/j.jad.2010.02.126

[40] Z. K. Nekouei , H. T. N. Doost , A. Yousefy , G. Manshaee , and M. Sadeghei , “The Relationship of Alexithymia With Anxiety‐Depression‐Stress, Quality of Life, and Social Support in Coronary Heart Disease (A Psychological Model),” Journal of Education and Health Promotion 3 (2014): 68. 25077161 10.4103/2277-9531.134816 PMC4113983

[41] N. H. Şahin , M. Güler , and H. N. Basim , “The Relationship Between Cognitive Intelligence, Emotional Intelligence, Coping and Stress Symptoms in the Context of Type A Personality Pattern,” Turkish Journal of Psychiatry 20 (2009): 243–254. 19757224

[42] P. Ramezannezhad , R. Rahmati , F. Zarimeidani , et al., “Personalized Patient Education: Patient's Perspective,” Iranian Biomedical Journal 28 (2024): 66.

